# Current trends of percutaneous nephrolithotomy in a developing country

**DOI:** 10.1590/S1677-5538.IBJU.2017.0292

**Published:** 2018

**Authors:** Carlos A. Batagello, Fabio Carvalho Vicentini, Giovanni Scala Marchini, Fabio Cesar Miranda Torricelli, Miguel Srougi, Willian Carlos Nahas, Eduardo Mazzucchi

**Affiliations:** 1 Divisão de Urologia, Grupo de Endourologia Hospital das Clínicas Faculdade de Medicina da Universidade de São Paulo, São Paulo, SP, Brasil

**Keywords:** Calculi, Nephrostomy, Percutaneous, Epidemiology

## Abstract

**Introduction:**

To present the current practice patterns on percutaneous nephrolithotomy (PCNL) in a developing country.

**Materials and Methods:**

A survey was offered to Brazilian urologists during the II International Endourology Symposium held in Sao Paulo, in 2015. The first seven questions were related to demographic data while the 20 remaining were directed to urologists who performed PCNL.

**Results:**

From 250 participants, 100 replied to the survey, 81% performed PCNL and 60.4% of performers had been in practice for less than 15 years. Eighty-one percent were trained in the prone position and 64% in supine. PCNL was learned during the residency in 66.7% and 2.5% had fellowship training. Prone position was the preferred decubitus for simple or complex calculi, though for obese patients there was no difference. Younger surgeons prefer supine while older surgeons prefer prone. The access was obtained by the surgeon in all cases, 96.3% use fluoroscopy and 3.7% prefer ultrasonography. Forty-seven percent use ultrasonic lithotripters and 4.1% laser. For kidney drainage, 71.6% place a nephrostomy tube. Double J stent is left in 77%. The postoperative image method was CT for 50%. Colonic injury was reported by 25%, predominantly in the senior group without statistically difference between positions.

**Conclusions:**

From a selected group of urologists, we observe that Brazilian urologists usually gain their own access for PCNL guided by fluoroscopy. They predominantly prefer the prone position, use fascial dilators, ultrasonic lithotripters and place a nephrostomy tube when exiting the kidney. Fellowship programs, ultrasonography, flexible nephoscopy and tubeless procedures could be encouraged.

## INTRODUCTION

Percutaneous nephrolithotomy (PCNL) has become the standard of care for large stones (1-3). Recent studies have demonstrated an increase in the rates of PCNL use, despite significant advances in ureteroscopic (URS) efficacy (4-6). However, this is not a worldwide phenomenon. Analyses from Canada, Australia and the UK suggest PCNL utilization has remained stable or decreased while use of URS technique continues to increase (5). In Brazil, between 1998 and 2012, PCNL had the highest relative increase followed by URS (7).

The growing prevalence of stone disease, reaching 7% in women and 10% in men in the USA (8), highlights the importance of a better understanding of current regional practices. In Brazil, the number of stone-related hospitalizations increased 15.7% from 1998 to 2012 (7). Sivalingam performed a survey of current practices in PCNL among members of the Endourological Society (9). No data is yet available for Brazil.

The aim of our study is to understand the current Brazilian practice patterns in PCNL and to explore different aspects of the technique and practice settings. This information will establish the first critical analysis of PCNL in Brazil, allowing for the delineation of regional treatment strategies and educational programs.

## MATERIALS AND METHODS

After Institutional Review Board approval, a questionnaire about trends of PCNL was offered to participants of the II International Endourology Symposium (Stone) held in São Paulo, Brazil, in September 2015. Urologists were invited to participate in the research and received a questionnaire consisting of 27 questions (Table-1). The anonymous survey collected demographic data in the first seven questions, answered by all urologists, while the 20 remaining questions were addressed to those who perform PCNL regularly. Years of practice, residence/fellowship training, patient decubitus preferences and other technical aspects were investigated.

**Table 1:** Questionnaire.

	Question	Options
**1**	How old are you? (years)	
**2**	What is your gender?	male female
**3**	How long ago did you finish your residency training? (years)	0 - 5 5 – 10 10 – 15 15 – 20 >20
**4**	Practice setting	Public hospital Private hospital Mix public and private hospital
**5**	What Brazilian Federal region are you from?	North Northeast Central West Southeast South
**6**	Do you perform PCNL?	Yes No
**7**	If no, why not?	No training No equipment No interest
**8**	Where did you learn to perform PCNL?	Urology residency Endourology fellowship With an expert At a congress/course
**9**	How many PCNL did you perform in the last 12 months?	0-6 7-12 13-24 25-50 >50
**10**	Who gain the renal access?	Urologist Radiologist
**11**	How is the renal access guided?	Ultrasound Fluoroscopy/retrograde pyelography Fluoroscopy/intravenous contrast Ultrasound and fluoroscopy
**12**	What image method is used for surgery planning?	Plain radiography Ultrasound Plain radiography + ultrasound UIV Non contrast CT Contrast CT
**13**	Are you trained for PCNL in the prone position?	Yes No
**14**	Are you trained for PCNL in the supine position?	Yes No
**15**	Are you trained for PCNL in the supine lateral position?	Yes No
**16**	What patient position do you prefer for a non-complicated case?	Prone Supine Lateral
**17**	What patient position do you prefer for obese patients	Prone Supine Lateral
**18**	What patient position do you prefer for a complex case? (Ex: staghorn)	Prone Supine Lateral
**19**	What is your preferred method for dilation?	Metallic (Alken) Fascial (Amplatz) Balloon Alken + Amplatz
**20**	What is your preferred method for stone fragmentation?	Pneumatic Ultrasonic Pneumatic + ultrasonic Laser
**21**	Have you had a colonic injury during PCNL?	Yes No
**22**	If you have had a colonic injury, how was the patient positioned?	Prone Supine
**23**	Do you usually finish the surgery performing flexible nephroscopy?	Yes No
**24**	How often do you use a nephrostomy tube?	Always uses Never uses Depending of the case
**25**	About ureteral drainage?	Ureteral Catheter Ureteral Stent (2J) Tubeless
**26**	What is your preferred postoperative imaging method?	Plain Radiograph Ultrasound Plain radiography + ultrasound UIV CT scan
**27**	What is your final impression about PCNL?	Like to perform Don't like to perform

Uncomplicated cases were defined as non--staghorn stones in a patient with no neurological bladder and without urinary diversions. Obesity was defined as BMI above 30Kg/m^2^. Complex cases were defined as staghorn calculi, abnormal anatomy, or urinary diversion. Regarding exit strategies, the use of nephrostomy tube, ureteral stent, ureteral catheter and tubeless technique were evaluated.

Statistical analysis was performed with SPSS software (version 16.0). Fisher exact test was used to compare categorical data. Student t--test was used to compare continuous data. The level of significance was defined as p<0.05.

## RESULTS

Of 250 participants, 100 replied to the survey and were included in the analysis. Eighty--one respondents (81%) performed PCNL regularly (Performers Group-PG). Nineteen urologists (19%) didn't perform PCNL (Non-Performers Group-NPG).

### Group characteristics

Table-2 outlines the demographics of both groups. The experience level was significantly different between them, with a greater proportion of the performers in practice for less than 15 years (60.4% vs. 31.6%; p=0.0259). Accordingly, PCNL performers were younger than non-performers (44.4 vs. 51.2 years; p=0.0253). There were no significant differences between the groups regarding gender, practice setting or the five Brazilian Federal Regions they were from. The main reasons reported by NPG for not incorporating PCNL into their practice were lack of training (60%), non-availability of equipment (20%), and lack of interest (20%).

**Table 2:** Patient demographics (all responders), performers and non-performers.

Characteristic		All responders	Perform PCNL		
	Group	(n)	Yes	No	*p*-value
Gender (n)	Female	3	2.5% (2)	5.3% (1)	0.4724
	Male	97	97.5% (79)	94.7% (18)	
Residency (years since completion of training)	0-5	28	33.3% (27)	5.3% (1)	0.0259
	5-10	12	12.3% (10)	10.5% (2)	
	10-15	15	14.8% (12)	15.8% (3)	
	15-20	10	11.1% (9)	5.3% (1)	
	>20	35	28.4% (23)	63.2% (12)	
Practice setting	Private Hospital	58	55.6% (45)	68.4% (13)	0.3014
	Public Hospital	12	11.1% (9)	15.8% (3)	
	Mix public private	30	33.3% (27)	15.8% (3)	
Region (n)	South	16	16% (13)	15.8% (3)	0.6569
	Southeast	61	63% (51)	52.6% (10)	
	West Central	12	9.9% (8)	21.1% (4)	
	Northeast	9	8.6% (7)	10.5% (2)	
	North	2	2.5% (2)	0% (0)	
Age in years (n)	<30	4	3.7% (3)	5.3% (1)	0.0054
	31 - 40	41	46.9% (38)	15.8% (3)	
	41 - 50	20	22.2% (18)	10.5% (2)	
	51 - 60	20	13.6% (11)	47.4% (9)	
	>60	15	13.6% (11)	21.1% (4)	
Age	Mean (SD)		44.4 (12.7)	51.2 (11)	0.0253

### Performers Group

Eighty-one urologists constituted the PG, male gender was predominant (97.5%) and 60.4% had finished their training program less than 15 years prior to responding to the questionnaire. The learning of PCNL occurred during residency for 66.7%, 17.3% were trained by other urologist with expertise in PCNL, 13.6% at a congress/course and 2.5% had a formal endourology fellowship. Forty-five performers (55.6%) worked in private practice while nine (11.1%) in public hospitals. Mixed public and private practice was represented by 27 Performers (33.3%). Two thirds were from the Southeast region of Brazil and 16% were from the South. Analyzing the number of procedures, 38.3% of the urologists performed up to six PCNL a year, 22.2% performed 7-12, 19.8% performed 13-24 and 9% each for 25-50 and >50PCNL a year.

### Technical aspects

Differences in operative procedures are listed in Table-3. Computed tomography (CT) was used pre-operatively by 75 Performers (92.6%) and half preferred non-contrast computed tomography (NCCT).

**Table 3:** Differences in operative procedures and surgeon preferences.

			N	%
Imaging studies preop		CT	75	92.6
Decubitus training		Prone	61	81,3
		Supine	48	64
		Lateral	11	14.7
**Decubitus preference**				
	Usual case	Prone/Supine/latera/other	46/29/0/0	61.3/38.7/0/0
	Complex calculi	Prone/Supine/Lateral/Other	45/27/3	60/36/0/4
	Obesity	Prone/Supine/Lateral/Other	36/34/2/3	48/45.3/2.7/4
**Renal access**				
	Performed	Urologist	81	100
		Fluoroscopy	78	96.3
	Imaging	Ultrasound	3	3.7
		Amplatz	37	88
	Method	Balloon	6	8.1
		Alken	6	8.1
		Amplatz + Alken	25	33.8
	Lithotripters	Ultrassonic	35	47.3
		Pneumatic	27	36.5
		Ultrassonic + Pneumatic	9	12.2
		Laser	3	4.1
	Kidney drainage	Nephrostomy	53	71.6
		Tubeless	21	28.4
		Ureteral stent (2J)	57	77
		Ureteral catheter	5	6.8
Flexible nephroscope		At the end of surgery	8	10.8
Postop imaging		CT scan	37	50
		Plain radiograph	15	20.3
		Ultrasound	5	6.8
		Plain radiograph + us	16	21.6

We obtained 75 responses regarding decubitus training. Sixty-one (81%) performed PCNL in prone position, 48 (64%) in supine and 11 (14.7%) in lateral. Uncomplicated cases and complex calculi were operated in the prone position by 46 (61.3%) and 45 (60%) of Performers, respectively. No difference in decubitus preference was observed for obese patients.

**Figure 1:**
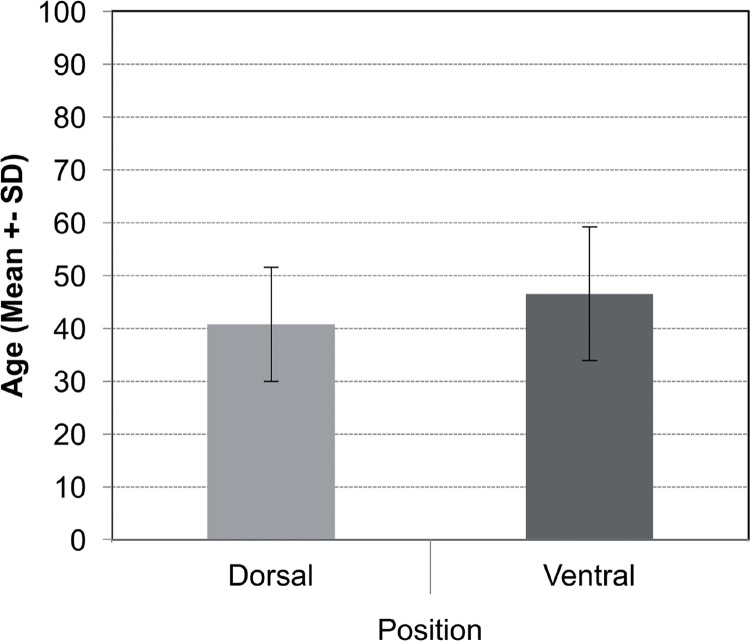
Mean age of the urologist according to the position preference during PCNL.

Comparing decubitus preference for uncomplicated cases we found that supine position was preferred by urologists with less than five years out of residency training (n=14;48.3%) while the prone was preferred by surgeons more than 20 years out of training (n=17; 37%) (p=0.0552). The urologists who preferred use of the supine position were primarily between the ages of 31-40 (n=19; 65.5%), while the urologists who preferred use of the prone were over 60 years of age (p=0.021). The mean age was statistically lower in the supine group (40.8 years±10.8) versus the prone (46.5 years±12.6) (p=0.039) (Figure-1).

The renal access was obtained exclusively by urologists, 78 (96.3%) used fluoroscopy and 3 (3.7%) preferred ultrasonography guided renal puncture. From questions 19-27 we obtained 74 responses. Half used amplatz fascial dilators (Cook Medical Inc., USA), while six (8.1%) preferred balloon. Alken dilators (Karl Storz, Tuttlingen, Germany) were used by six (8.1%) and Alken plus Amplatz by 25 (33.8%). Thirty-five (47.3%) surgeons used ultrasonic lithotripters, 27 (36.5%) pneumatic devices, nine (12.2%) combined ultrasonic/pneumatic and three (4.1%) laser. From 74 Performers, eight (10.8%) carry out flexible nephoscopy at the end of the procedure looking for residual fragments not detected by fluoroscopy. For kidney drainage, a nephrostomy tube was used by 53 (71.6%) while 21 (28.4%) preferred tubeless technique. Ureteral stent was left by 57 (77%), while 5 (6.8%) preferred a ureteral catheter.

Post procedural imaging method was CT for 50% of responders. KUB was used by 20.3%, ultrasonography by 6.8% and ultrasonography plus KUB by 21.6%.

### Complications

Data on postoperative complications, in particular colonic injury, was collected. Of 81 performers, we recorded 74 responses, with 19 (25.6%) reporting this complication at least once in their practice. Of these nineteen, 9 reported this lesion in the supine position (47.4%, 95% CI 27.3-68.3) and 10 in the prone (52.6%, 95% CI 31.7-72.7).

Colonic injury and years of practice were correlated and we found it happened in 2 urologists (10.5%) with up to 5 years of practice and in 9 (47.4%) with more than 20 years (p=0.015).

### Surgeon opinion about PCNL

When asking the urologists who perform PCNL their opinion about the procedure, 11 (14.9%) didn't like to perform it, showing the difficulties regarding technique and complications.

## DISCUSSION

Stone disease prevalence is increasing worldwide (2) and highlights the importance of better understanding of current local and global practices. For large calculi, The European Association of Urology (EAU) and The American Urological Association (AUA) guidelines recommend PCNL as the first-line treatment (2, 3, 10).

However, PCNL utilization has varied across the world. In the USA and UK studies have demonstrated an increase in the annual rate of PCNL (8, 9, 11) while in Australia, despite an increase in the total number of all stone-related procedures, the proportion represented by PCNL had decreased (6, 11). In Canada, PCNL use has remained constant (11). Marchini et al. performed an analysis of urolithiasis tendencies in Brazil revealing an increasing number of surgical procedures over the last 15 years, with PCNL showing the highest relative increase (7).

In this study, we investigated PCNL aspects and trends in a select population of Brazilian urologists attendees of an endourological symposium. It is the first PCNL demographic study in Brazil. Note that most of the participants were from the South or Southeast regions, notably the most economically developed. A more representative population of all urologists across the regions could show geographic differences.

When comparing PG with NPG, we observed different experience levels, with a greater proportion of PG in practice <15 years and significantly younger than NPG showing that PCNL was recently incorporated into the Brazilian urologist's practice and is preferentially performed by younger urologists that had PCNL training in their residency.

Analysis from the NPG showed that the main reason for not performing PCNL was lack of training and more than half of the responders weren't trained in PCNL during their residency program. Learning of PCNL occurred during residency for 66.7% while only 2.5% had formal fellowship, reveling a lack of endourology fellowship programs in Brazil. These responses emphasize the importance of increasing educational programs as well as investments in teaching centers.

We observed that the PG was mainly from the South and Southeast regions, half were private practice urologists and one third had mixed public and private practices. Examining the number of procedures in a year, 38.3% performed up to six PCNL while 9.9% performed 25-50PCNL. Therefore, urologists had a low-volume of cases, with only 10% of them performing an adequate number of procedures. Multiple reports have confirmed a volume-outcome relationship with PCNL and demonstrated that hospitals that perform >33PCNLs per year have significantly shorter length of stay and lower complication rates (12-14).

Regarding training and patient decubitus, 81% of performers were trained to obtain renal access in prone position and 64% in supine. Although prone has been the preferred position for PCNL for decades, the supine decubitus position (15, 16) is becoming more popular among responders of our survey which contrasts with CROES data, that shows it is currently used in only 20% of centers worldwide and its practice is almost null in North America and Australia (11, 16). The prone position was preferred for both usual (61.3%) and complex cases (60%).

Comparing position preferences with years of practice, we found that less experienced surgeons (<5 years of practice) preferred the supine position (48.3%) while more experienced surgeons (>20 years) preferred the prone position (37%) (p=0.0552). The supine position was preferred by urologists from 31-40 years of age (p=0.021).

Decubitus preference in obesity was also investigated. Overall, obese patients have similar outcomes when compared to the general population, except for super-obese (BMI>40) who have a higher chance of more severe complications (17). Unlike the results of the CROES study, suggesting that supine position was utilized significantly less often in the obese cohort (17), we found no significant difference in decubitus preference.

After selection of position, obtaining safe access is one of the most important step of PCNL. According to Sri and colleagues (9), 77% of responders established their renal access and the fellowship training was a significant determinant for it. Our findings confirmed this trend. The renal access was obtained exclusively by urologist and 96.3% preferred fluoroscopy guided renal puncture, while 3 (3.7%) used ultrasound. Accordingly, CROES data reveals that in PCNL 86.3% of patients had fluoroscopic guided access versus 13.7% with ultrasound guidance (18). This difference indicates that training programs for ultrasound guided access for PCNL should be encouraged.

Eighty-eight percent used Amplatz fascial dilators while 8.1% preferred balloon, correlating with the results of the CROES study that show preference for serial dilatation in Asia and South America and the predominant use of balloon dilatation in North America (19). This fact can be explained in part by its higher costs and possibly a lack of training.

PG preferred ultrasonic lithotripters (47%) over pneumatic (36.5%), in contrast to the CROES data which states that pneumatic lithotripters were used more frequently, followed by ultrasonic-only, combination ultrasonic/pneumatic and finally laser lithotripters (20). Nephrostomy tube was used by 71.6%, similar to the trend reported by CROES (91.2%) and Sivalingam et al. (76%) (9, 21), but tubeless technique was used by 28.4%, even though many trials (11, 22) have confirmed that in selected cases the tubeless technique can be safe (23). A ureteral stent was left in 77% of cases and a ureteral catheter in 6.8%.

In addition to high-resolution fluoroscopy and postoperative CT, routine use of flexible nephoscopy at the end of the procedure can maximize stone clearance. In our survey, 11% performed it at the end of surgery and NCCT was the preferred postoperative imaging method for 50%.

Colonic injury, reported in less than 1% of cases, has great significance due to its diagnostic challenges as well as its outcome (24). In our study, colonic lesion occurred predominantly in the senior urologists group (p=0.015) with no difference in decubitus positioning, the same as reported by Wu et al. (25). Finally, our questionnaire revealed that 15% of PCNL performers don't enjoy performing the procedure. This calls attention to the technical difficulties and potential complications.

Our study is not without limitations. It evaluated the practice of a limited group of urologists interested in endourology, mainly from the most developed regions of Brazil. Important practice differences could possibly be observed in a more geographically representative sample of Brazilian urologists. Our response rate was relatively low, despite being higher than that obtained by other studies. Nevertheless, we believe the information from our survey is novel and can assist in developing educational programs for PCNL in Brazil and other similar countries worldwide.

## CONCLUSIONS

From a selected group, we observed that Brazilian urologists usually gain their own access for PCNL guided by fluoroscopy. They predominantly prefer the prone position, use fascial dilators, and place a nephrostomy tube when exiting the kidney. The supine decubitus is gaining preference among young Brazilian urologists. Fellowship programs, use of ultrasonography, ultrasonic lithotripters, flexible nephoscopy and tubeless procedures should be emphasized as goals for PCNL practice.
